# Ethical Dilemmas with Regard to Elderly Patients with Hip Fracture: The Problem of Nonagenarians and Centenarians

**DOI:** 10.3390/jcm11071851

**Published:** 2022-03-27

**Authors:** Mario Herrera-Pérez, David González-Martín, Emilio J. Sanz, José L. Pais-Brito

**Affiliations:** 1 Department of Orthopaedics, University Hospital of Canary Islands, 38320 Tenerife, Spain; herrera42@gmail.com (M.H.-P.); paisbrito@gmail.com (J.L.P.-B.); 2 Department of Surgery, School of Medicine, Universidad de La Laguna, 38320 Tenerife, Spain; 3 Department of Pharmacology, School of Medicine, Universidad de La Laguna, 38320 Tenerife, Spain; esanz@ull.es

**Keywords:** autonomy, capacity, clinical ethics, informed consent, legal aspects

## Abstract

Hip fracture is the most feared complication of osteoporosis, producing up to 30% mortality at the first year. With the aging of society, it is increasingly common to deal with ethical dilemmas that involve decision making in the elderly patient with a hip fracture. The objectives of the present work are to describe the main bioethical dilemmas in this group of patients and their relationship with surgical delay. We conducted a retrospective descriptive study that studied an elderly population admitted to a University Hospital with a diagnosis of hip fracture. In total, 415 patients were analyzed. The majority received surgical treatment, a correct application of the principles of justice, non-maleficence and beneficence is verified, but a possible violation of the principle of autonomy is confirmed. Based on the results of this study, the elderly population may somehow lose their principle of autonomy when they enter a hospital due to a hip fracture. On the other hand, the so-called ageism due to ignorance can influence the surgical delay and therefore the mortality of these patients.

## 1. Introduction

Ageing populations are a global phenomenon with important biopsychosocial and economic impacts that will affect all areas of our lives. The global population over 60 years of age will increase from 900 million in 2015 to 2000 million in 2050, according to the World Health Organization (WHO) [1,2]. From a musculoskeletal perspective, old age means a higher level of osteoporosis, a silent, asymptomatic disease that produces bone fragility and when it manifests as fractures it can put the health of sufferers at risk. Of the various osteoporotic fractures, hip fractures are the most likely to cause mortality: the increased mortality in patients after a hip fracture compared with controls varies between 8% and 36% per year [1,3,4,5,6]. Hip fracture in the elderly represents approximately 40% of traumatology admissions in the developed world [1]. Therefore, because of its prevalence and clinical significance, especially in terms of mortality, it is an especially important area in biomedical traumatology research [2]. In the case of an elderly patient, over 85–90 years of age, with a hip fracture, the question arises as to whether it is ethically acceptable to subject the patient to surgery, assuming the risks (anesthesia, blood loss, convalescence) as opposed to the conservative attitude of no surgical intervention. It should be borne in mind that the patient is often vulnerable, not only because their advanced age may prevent them from being self-sufficient when it comes to daily living activities (walking, reading, seeing, hearing, etc.), but also because on many occasions there is an associated deterioration in mental function that hinders both the doctor–patient relationship and the decision-making process, which may be encompassed within the principle of autonomy [7]. All these circumstances described determine that, in many cases, the admitted elderly patient is considered ineligible to receive information, regardless of the assessment of their mental capacity to receive this, with the typical circumstance being arrived at where the family is informed before the patient, who should be the holder of the information. This is what many authors have called the tacit pact of silence or conspiracy of silence [8]. In this pact, the influence of ageism is crucial.

### 1.1. Ageism

This term was coined in 1968 by the gerontologist and psychiatrist Robert Butler, to refer to discrimination against older people, based on the terms sexism and racism [9,10]. Butler defined “ageism” as a combination of three connected elements. These include harmful attitudes towards older people, old age, and the ageing process; discriminatory practices against older people; and institutional practices and policies that perpetuate stereotypes about older people [11]. The fear of death and the fear of disability and dependency are the leading causes of ageism. In the social and health care system, ageism often manifests as ill-treatment, a lack of attention, and even the restriction of access to specific resources, as discussed later [9,10,11].

### 1.2. Legal Aspects

Elderly patients, regardless of their mental and physical capacities, have certain inviolable rights registered by multiple international treaties. Within this subsection, we consider three rights that should be considered. However, these are not the only ones (because there are also many others, such as confidentiality, as well as those contained in the Code of Medical Ethics): the right to non-discrimination (by age in this case), the right to accurate and truthful information, and the right not to receive treatment [12,13,14].

### 1.3. Bioethical Aspects of Caring for Elderly Patients

Clinical care and clinical ethics must be incorporated into geriatric care in order to develop high-quality care. Concerning the care of the elderly with hip fracture, the creation of orthogeriatric units has been a genuine revolution in the overall treatment of this, particularly vulnerable patients [15]. Incorporating values, together with objective facts, ensures that this care preserves the dignity of every elderly patient who receives health or social care. However, we are currently in a situation of moral pluralism in which it is not easy to reach agreements on what should and should not be carried out in the care of the elderly. Several methods have been developed in the field of bioethics that can help determine the ethical minimums required of any individual (professional, volunteer, politician, manager, and others.) about the care of the elderly [16]. Within the different methods that exist in the field of bioethics, and although the same problem can be approached from various perspectives, we consider principialism, based on the principles described in Beauchamp and Childress [16], and specifically that modified by Professor Diego Gracia Guillén, known as the “deliberative method”, to be appropriate for the topic we are dealing with here: an elderly patient admitted to hospital which must be assessed for suitability for surgery [17,18].

### 1.4. Hip Fracture in the Elderly

In the developed world, it is also the most critical complication of osteoporosis in terms of mortality, morbidity, and cost [1,19]. Globally, there were around 1.3 million hip fractures in 1990, and according to estimates, there could be as many as 20 million cases in 2050 [20,21,22]. The mortality associated with hip fracture is 5–10% in the first month, 4% in-hospital mortality, and 30–33% in the first year after the fracture (although this is somewhat higher in men than women), figures that exceed those of the mortality due to colon, breast, or prostate cancer for the same age group. This mortality is related to respiratory complications, ischemic heart disease, and heart failure [23]. In total, 50% of patients do not recover the functional level they had before the fracture, close to 25% need care for long periods, and 20% have ongoing dependency: ultimately, only 5% return to their prior functional status [19,24].

It is expected that the global incidence of hip fractures will increase markedly in the coming decades [20,25,26]. Currently, according to the International Osteoporosis Foundation (IOF), around the world each year there are close to 9 million new osteoporotic fractures, of which 1.6 million are hip fractures; the forecasts for the year 2050 ranging from 4.5 to 6.3 million. This increase will be widespread, but especially dramatic in Asia. The current figure of hip fracture incidence in Europe comes from the report of Johnell and Kanis: they estimated that in Europe in the year 2030, around 1,000,000 hip fractures were suffered by men and women, making Europe the region with the highest number of hip fractures in the world, with 38% of the global total [27,28,29]. The expected increase in Europe is around 135% over the next 50 years, involving an estimated figure of close to a million new hip fractures a year. The morbidity and mortality rates are high, and this condition generates many disabilities, extended stays in chronic centers, and a considerable deterioration in the quality of life of the sufferer. If in 2000, 25,000 hospital beds were needed for treatment (0.88% of those available), in 2010 it was predicted that this number would be 30,000 (1.06% of those available), and by 2050 this figure would have almost doubled, meaning 2% of the available beds could be filled by hip fracture sufferers [24].

The main objectives of this work are:

(1) To describe the most frequent bioethical conflicts relating to elderly patients admitted to hospital with a hip fracture.
(2) To provide suggestions to help resolve the conflicts encountered.

## 2. Materials and Methods

Retrospective descriptive study of the main bioethical conflicts of patients admitted with a diagnosis of hip fracture in a university hospital from January 2017 to January 2019.

The inclusion criteria were:

- Hip fracture.
- Patients aged more than 65.

The exclusion criteria were:

- Inability to access medical history data (patient referred from another center).

For this study, we used the electronic medical record review from the orthopedic department from the past two years, on the medical practice carried out in the center regarding these patients. A search was made on the SAP system without providing any confidential patient data, collecting the following parameters:

- Age.
- The presence of dementia.
- Anesthetic risk according to the American Society of Anesthesiologists (ASA) score that distinguish between ASA I to ASA V, from lowest to highest risk) [30].
- Surgical delay and cause of that delay.
- Cause of non-surgical indication.

## 3. Results

Between January 2017 and February 2019, a total of 415 patients with a diagnosis of hip fracture were admitted to hospital (Table 1). Of these, 75% were women, with an average age of 88.2 (62–102 years), and 25% were men, with an average age of 84.3 (67–98 years). For the purposes of differentiated analysis, the series was split into three age groups: <90 years of age: 382 cases; 90–100 years of age: 25 cases; >100 years of age: 8 cases. The patients predominantly came in from their habitual residence (70%) but with nuances; in the age 90–100 group, all except one lived in a nursing home (24 cases), as did 6 of the centenarians. Dementia was present in 40% of the series, also differing according to age: 80% of patients older than 90 had mental deterioration. However, only 20% of our series (81 patients) were legally incapacitated.

The delay to surgery was 3.6 days (1–17) and, in most cases, this was, firstly, due to the lack of availability of an operating room, and secondly, to optimize the medical care of the patients prior to surgery (Table 2). In 10% of the cases (42 patients), the surgical delay was longer than 48 h due to family members disagreeing about the suitability of surgical treatment for their relative (40 cases), and in 2 cases due to disagreements with anesthesiology. Of the entire series, only 18 patients did not undergo surgery (4.5% of the total), meaning 95.5% of the cases opted for an operation. The causes of non-surgical intervention were:

- Nine cases: worsening of the general state of health and death in the hospital environment.
- Five cases: joint decision made by the family and geriatrician due to the poor medical status at admission, tolerating conservative treatment and being discharged.
- Four cases: refusal of the family to assume the treatment risk (4 patients with advanced senile dementia aged 91, 89, 93, and 96), all of whom were discharged.

Regarding informed consent for surgery, for 55% of the patients in this series, the IC was signed by a relative or legal representative (100% of centenarian patients and 80% of nonagenarians), with the remaining 45% signed by the patients themselves.

## 4. Discussion

Health-related ethical conflicts are a source of concern for many healthcare professionals involved in social and health care. An elderly patient with a hip fracture is an example of a vulnerable patient, not only because of the frequent associated comorbidity and sensory deficits, but also because of the aggravated situation caused by the fracture: pain and the need for hospital admission with the so-called loss of reference (loss of contact with family, caregivers, and the usual environment). These are, therefore, patients that we must protect in the broadest sense of the word. The four principles articulated in 1979, in the Belmont Report, were a milestone in medical practice. When making the most appropriate treatment decisions for an elderly patient with a hip fracture, the principles of non-maleficence, which ensure the life of individuals as a guarantee that they will not be harmed, either by the execution of a harmful action or by omission of an action due to avoiding harm, and the principle of justice, which ensures non-discrimination and equal access to social goods and resources (health in this case), come into play.

Regarding the results obtained in our study, it is noteworthy, in relation to the principle of autonomy, that in a high percentage of cases (55%) the patient was not correctly informed (or, at least, this is not recorded), and it was decided that the IC should be signed by relatives or representatives. If we subtract from this percentage those patients with dementia (40%), in at least 15% of the cases (60 patients) the decision to authorize surgery was not made by the patient. This is only legally admissible when the patient has impaired abilities. This is affirmed by Article 5.3 of the Law of Patient Autonomy [13] “where, in the opinion of the attending doctor, the patient lacks the capacity to understand the information because of their physical or mental condition, the information will be brought to the attention of persons that have family or de facto connections with them.” However, this clarification is not found in the medical history of any of these 60 patients, which may be legally objectionable. This violation of the principle of autonomy in some cases also represents a paternalistic attitude on the part of the health professional, in contrast to the desirable participative decision-making model, since the patient must be correctly informed of their process, and this can only be explained by a flagrant lack of attention and respect for the dignity of the patient, since it was undoubtedly more comfortable for the treating physician to talk to the relatives (generally younger than the patient), and not make an effort to obtain authorization for surgery from the patient themselves, ignoring the fact that the vulnerability of a patient affected by illness does not deprive them of their personal autonomy and of their obligation to personally manage their own life and decisions. Regarding surgical delay, one of the main causes of mortality, even though the main reason for this was a lack of material resources (availability of the operating room), 10% of cases were due to ageism or age discrimination, since family members were not aware that surgery is indicated in almost 95% of cases. However, this may be better defined as ageism due to ignorance, as it is undoubtedly a lack of information on the suitability of surgical treatment despite age that made them doubt the pertinence of an operation.

A very interesting aspect to consider in elderly patients is the true concept of “old age”—should we consider a patient to be elderly when they are older than 80, 90, or 100 years of age? With the advances in multidisciplinary patient care after admission for hip fracture, their ability to withstand surgical aggression is increasing, with the geriatrician playing a key role in optimizing patients prior to surgery, leading to the momentum built in this field over recent years, and the creation of specialized orthogeriatric units [4,5,15,31,32]. Special mention should be made about the subgroup of nonagenarian patients, who are becoming increasingly frequent (25 cases in this series), as this subgroup is particularly critical due to the mental deterioration and the high medical comorbidity that is often present. Some authors state that the main criterion to follow in order to assess surgical treatment in patients over 90 years of age should be their mental state and their previous mobility. In 2005, Ooi et al. published an interesting study comparing patients over 90 years of age admitted for hip fracture, some treated surgically and others conservatively, including a 2-year follow-up period. They observed equally high mortality figures (49%) in both groups, whether they had been operated on or not; however, the same authors acknowledge that nonagenarian patients who had received surgery had a significantly increased walking capacity compared to those treated conservatively [33]. The first author to pose the dilemma of whether to operate, or not, on very elderly patients, i.e., nonagenarians, was Jennings in 1999, who, after studying a cohort of 50 hip fracture patients aged more than 90, concluded that although up to 50% of cases presented some type of postoperative complication, the majority of patients studied achieved the same mobility level they had prior to the fracture, and, therefore, considered that this type of surgery was equally beneficial in this age subgroup [34]. Along the same line, more recently an interesting article was published comparing the morbidity and mortality associated with hip fracture in nonagenarian and centenarian patients. No statistically significant differences were found between the two groups, so the authors do not consider advanced age as a factor of poor prognosis per se; however, they do place emphasis on the prior medical status of these patients [35].

The current problem is that the number of nonagenarian patients with hip fractures is still on the rise, and even more seriously, it is already relatively common to treat centenarian patients with hip fractures. In this sense, in 2004, authors from a public hospital in Edinburgh published the most complete study to date on the increased mortality in centenarian patients [36]. Comparing two groups of patients, 18 elderly patients aged over 100, with another 18 aged between 75–84 with similar levels of comorbidity, they reached the conclusion that the centenary group had a minimal increased mortality (51% versus 48%) although the percentage of post-surgery disability was higher in younger patients, perhaps because the centenarians already had more limited mobility. The presence of medical comorbidities in people with hip fractures is frequent. In a recent article, the main cause recorded for surgical delay was the presence of comorbidities that required the patient to be stabilized; the second was the presence of anticoagulation and antiaggregation [37,38,39]. In none of the articles we reviewed did we find that age is a negative factor when it comes to early surgery, as most authors recommend surgery in the first 48 h to avoid the appearance of possible complications [32,35,37].

Finally, the presence of some degree of senile dementia, present in 40% of our series, deserves separate consideration. Studies have shown that patients with dementia are at an increased risk of sustaining a hip fracture and tend to have worse functional outcomes than those who do not suffer this condition. Baker et al. recently presented an interesting article in which they analyzed the decision-making process in this particularly vulnerable group [15]. The authors conclude that the participation of geriatricians in this decision-making process is crucial when assessing the fragility of these patients, as part of the multidisciplinary team including orthopedic surgeons, anesthesiologists, nurses, and social agents, as occurs in our center [40,41,42].

There are limitations and strengths in our study that should be acknowledged. The main methodological limitation of this study is that it is a descriptive retrospective study. This is a single-center study, so the percentages described may not be representative of other centers. Its main strengths are the broad sample studied and the improvement actions proposed by the authors.

Based on the results of this study, the elderly population may somehow lose their principle of autonomy when they enter a hospital due to a hip fracture. On the other hand, the so-called ageism due to ignorance can influence the surgical delay and therefore the mortality of these patients. Finally, the authors consider that some proposals should be take into account when treating a frail and ancient population:

- Essential training in bioethics for postgraduates.
- Updating of the same staff, including knowledge of the basic legal framework that regulates patient rights.
- Dissemination of information to civil society on the reality of hip fractures to emphasize the seriousness of this genuine epidemic.

## 5. Conclusions

Based on the results of this study, the elderly population may somehow lose their principle of autonomy when they enter a hospital due to a hip fracture. On the other hand, the so-called ageism due to ignorance can influence the surgical delay and therefore the mortality of these patients.

## Figures and Tables

**Table 1:** Demographics.

Hip Fractures (*n* = 415)	
Sex	Men 104 (25%) Women 311 (75%)
Age	Men 84.3 years (R 67–98) Women 88.2 years (R 62–102)
Age groups	<90 years→382 patients 90–100 years→25 patients >100 years→8 patients
Dementia	No 249 (60%) Yes 166 (40%)
Legal incapacity	No 334 (80%) Yes 81 (20%)
Comorbidities	≥3→100 (24%) Arterial hypertension→253 (61%) Diabetes→170 (41%) Dementia→166 (40%)

**Table 2:** Results.

Results	
Surgical treatment	No 18 (4.5%) Yes 398 (95.5%)
Surgical delay	3.6 days (R 1–17)
Surgical delay causes	(1) Availability of operating theatres→116 (28%) (2) Medical optimisation→91 (22%) (3) Other (10%): Family discrepancy→40 (9%) Anesthesia discrepancy→2 (1%)
Informed consent Signatory	Patient 187 (45%) Relative or legal representative 228 (55%)
